# Modulation of Microtubule Dynamics Affects *Brucella abortus* Intracellular Survival, Pathogen-Containing Vacuole Maturation, and Pro-inflammatory Cytokine Production in Infected Macrophages

**DOI:** 10.3389/fmicb.2017.02217

**Published:** 2017-11-14

**Authors:** Juliana Alves-Silva, Isabela P. Tavares, Erika S. Guimarães, Miriam M. Costa Franco, Barbara C. Figueiredo, João T. Marques, Gary Splitter, Sergio C. Oliveira

**Affiliations:** ^1^Departamento de Bioquímica e Imunologia, Universidade Federal de Minas Gerais, Belo Horizonte, Brazil; ^2^Department of Pathobiological Sciences, University of Wisconsin-Madison, Madison, WI, United States

**Keywords:** *Brucella abortus*, microtubules, TcpB, innate immunity, bacterial pathogenesis

## Abstract

The microtubule (MT) cytoskeleton regulates several cellular processes related to the immune system. For instance, an intricate intracellular transport mediated by MTs is responsible for the proper localization of vesicular receptors of innate immunity and its adaptor proteins. In the present study, we used nocodazole to induce MTs depolymerization and paclitaxel or recombinant (r) TIR (Toll/interleukin-1 receptor) domain containing protein (TcpB) to induce MT stabilization in bone marrow-derived macrophages infected with *Brucella abortus*. Following treatment of the cells, we evaluated their effects on pathogen intracellular replication and survival, and in pro-inflammatory cytokine production. First, we observed that intracellular trafficking and maturation of *Brucella*-containing vesicles (BCVs) is affected by partial destabilization or stabilization of the MTs network. A typical marker of early BCVs, LAMP-1, is retained in late BCVs even 24 h after infection in the presence of low doses of nocodazole or paclitaxel and in the presence of different amounts of rTcpB. Second, microscopy and colony forming unit analysis revealed that bacterial load was increased in infected macrophages treated with lower doses of nocodazole or paclitaxel and with rTcpB compared to untreated cells. Third, innate immune responses were also affected by disturbing MT dynamics. MT depolymerization by nocodazole reduced IL-12 production in infected macrophages. Conversely, rTcpB-treated cells augmented IL-12 and IL-1β secretion in infected cells. In summary, these findings demonstrate that modulation of MTs affects several crucial steps of *B. abortus* pathogenesis, including BCV maturation, intracellular survival and IL-12 secretion in infected macrophages.

## Introduction

The innate immune response is the first line of defense against pathogens. Immune cells sense pathogens through a set of evolutionary conserved pattern recognition receptors (PRRs). Upon recognition of pathogen-associated molecular patterns (PAMPs), some PRRs trigger an inflammatory response, producing cytokines and antimicrobial intermediates leading to the efficient destruction of invading pathogens (Medzhitov and Janeway, 2000; Akira et al., 2006). *Brucella abortus* is a facultative intracellular coccobacillus that causes brucellosis both in humans and cattle and leads to serious economic and public health burden in endemic areas (Pappas et al., 2006). Infection by *B. abortus* causes abortion and sterility in animals; whereas in humans, the disease is characterized by acute inflammation, undulant fever, endocarditis, arthritis, among other pathological manifestations, with approximately half a million new cases occurring each year worldwide (Pizarro-Cerda et al., 1998; Boschiroli et al., 2001; Yang et al., 2013).

Like several bacteria that have undergone a long evolution within mammalian hosts, *Brucella* evolved mechanisms to subvert cell immunosurveillance that ensures bacteria survival, proliferation and persistence within the host (Barquero-Calvo et al., 2007). In this regard, *Brucella* virulence is crucially dependent on its intracellular life cycle. Upon entry into host cells, *Brucella* resides within a membrane-bound compartment, the *Brucella*-containing vesicle (BCV) whose trafficking is controlled by the bacterium (Pizarro-Cerda et al., 1998; Comerci et al., 2001; Delrue et al., 2001; Salcedo et al., 2008; Starr et al., 2008). BCV suffers a maturation process characterized by limited interaction and acquisitions of the endoplasmic reticulum (ER) membrane until it is converted in a organelle competent to sustain bacterial replication. The BCV acidifies and acquires late endosomal marks such as Rab7 and LAMP-1 (Pizarro-Cerda et al., 1998; Celli et al., 2005). This vesicular traffic is largely performed by molecular motors on microtubules (MTs), essential components of the eukaryotic cytoskeleton that are implicated in important functions as cell division, migration, and intracellular signal transduction. Likewise, stabilization and destabilization of MTs directly affects cellular signaling and several signal transduction pathways (Gundersen and Cook, 1999).

Furthermore, bacterial pathogens deliver several effector proteins into host cells for modulating MT cytoskeleton dynamics (Yoshida and Sasakawa, 2003). *B. abortus* encodes a TIR domain-containing protein, TcpB (also termed BtpA) that co-localizes with MTs (Radhakrishnan et al., 2009) and increases the rate of nucleation as well as the polymerization phases of MT formation, acting as a stabilization factor that modulates MTs dynamics (Radhakrishnan et al., 2011). The MT stabilization properties of TcpB are attributed to the BB-loop region of the TIR domain, whereas a BB-loop mutant TcpB exhibits defective MT binding and stabilizing properties (Radhakrishnan et al., 2011).

In this study, we used nocodazole, paclitaxel and recombinant (r) TcpB as MT modulating agents. The complexity of brucellosis pathogenesis emphasizes the importance in understanding the mechanisms governing its intracellular trafficking and life cycle. Therefore, we investigated the effect of MT destabilization and stabilization on the intracellular trafficking of *B. abortus* as well as host innate immune responses.

## Materials and Methods

### Mice

C57BL/6 mice aged 6–8 weeks were used in this study. Animals were purchased from the animal facility at Federal University of Minas Gerais (UFMG). All the animal experimental procedures were pre-approved by the Institutional Animal Care and Use Committee of UFMG (CETEA #128/2014).

### Bacterial Strain

A variation of *B. abortus* virulent strain S2308 which constitutively expresses GPF (*B. abortus*-GFP) from our laboratory collection was used in the present study. Bacteria were grown overnight in Brucella broth medium (BD Biosciences Pharmingen, San Diego, CA, United States) at 37°C under constant agitation, before being used for macrophage infection. All work with *Brucella*, including animal experiments, was conducted in the biosafety cabinet or a primary containment device within a dedicated laboratory. Appropriate laboratory coats, gloves, and protective eyewear were provided to ensure the safety of personnel. All personnel working in the biosafety laboratory were trained and approved for entry by an individual knowledgeable in the biosafety practices.

### Pharmacological Reagents

Nocodazole and Paclitaxel were purchased from Sigma–Aldrich, Co. (St. Louis, MO, United States). Stock solutions of 5 mM were prepared for each reagent in dimethyl sulfoxide (DMSO) and kept frozen (-20°C). Further working dilutions were prepared in DMEM medium on the day of the experiments and used fresh.

### TcpB Expression and Purification

The expression plasmid containing the coding sequence for the TIR domain-containing protein (TcpB/BtpA) pD444-NH::TcpB was purchased from DNA 2.0 Inc., United States^1^, and the expression and purification of TcpB protein performed as previously described (Goncalves de Assis et al., 2015). A poly-lysine column (Thermo Fisher Scientific) was used for endotoxin (LPS) removal according to the manufacturer’s instructions.

### BMDM Culture

Bone marrow-derived macrophages (BMDMs) were obtained from C57BL/6 mice as previously described (Gomes et al., 2016). Briefly, bone marrow cells from each femur and tibia was flushed with 5 ml of Hanks’ balanced salt solution (HBSS). The resulting cell suspension was centrifuged, and the cells were resuspended in Dulbecco’s modified Eagle’s medium (DMEM; Gibco) containing 10% fetal bovine serum (FBS; Gibco), 1% penicillin-streptomycin, 1% HEPES, and 10% L929 cell-conditioned medium (LCCM) as a source of macrophage colony-stimulating factor (M-CSF). Cells were then plated into 10 cm cell culture dishes and cultured for 10 days in 5% CO_2_ atmosphere at 37°C. Four days after seeding, additional 10% LCCM was added to the culture, and on day 7 the medium was replaced by fresh differentiation medium. On the 10th day, differentiation into macrophages was complete, and the cells were released from the dish (using cold PBS) to be re-plated onto 24-well plates for infection. BMDMs were seeded at 5 × 10^5^ cells/well for cytokine secretion and colony forming unit (CFU) analysis, and at 1 × 10^5^ cells/well over sterile 12 mm round glass coverslips for microscopic analysis.

### Infection with *B. abortus*

Bone marrow-derived macrophages were infected for 1 h with *B. abortus*-GFP (MOI 200:1) as follows: prior to being infected, cells were washed once with PBS to remove traces of antibiotics from the differentiation media. Three hundred microliters of DMEM (supplemented with 1% FCS) containing bacteria was added to each well, and the cells were centrifuged at 600 *g* for 10 min before being kept at 37°C for an additional 50 min. At the end of the 1-h period cells were washed once with PBS to remove excess bacteria and media containing nocodazole, paclitaxel or DMSO (controls) were added at different concentrations (see section Results) for a further 4 or 24 h. At the end of the experiments, supernatant was collected for further analysis of cytokine secretion or the cells were fixed in 4% paraformaldehyde for immunofluorescence. For experiments with recombinant (r) TcpB, cells were pre-treated with 1, 5, or 10 μg/mL of rTcpB protein, with no detectable LPS, for 2 h prior to being infected with *B. abortus* (MOI:200) as described above. Cells were then washed once with PBS and cultured in medium containing TcpB (1, 5, or 10 μg/mL) for further 24 h.

### Immunofluorescence Microscopy

Infected BMDMs seeded onto glass coverslips and subjected to different treatments, as described above, were fixed in 4% paraformaldehyde, pH 7.4, at room temperature for 30 min. After fixation, cells were washed 3× with PBS and permeabilized with 0.1% saponin for 30 min. Cells were then washed 3× with PBS and incubated overnight with 1:250 rabbit anti-LAMP1 (Cell Signaling) or with 1:500 mouse anti-tubulin. Three washes with PBS were again performed prior to incubation for 1 h with 1:400 anti-rabbit Alexa 546 or 1:400 anti-mouse Alexa 488 secondary antibody. Cover-slips were mounted on slides using ProLong Gold antifade reagent with DAPI (Invitrogen). Images were collected using a Nikon C2 confocal laser microscope. The number of intracellular GFP-expressing bacteria and the percentage of *B. abortus* BCVs containing LAMP-1 were quantified at 24 h using the confocal microscopy images. Images from at least 85 infected cells documented from 3 independent experiments were used for the analysis (see figure legends).

### BMDM Viability Assay

The toxicity of nocodazole and paclitaxel treatment was evaluated in live/dead cell viability assays. BMDMs seeded on cover-slips and infected with *B. abortus* (as described above) were treated with 0.5 or 5 μM of nocodazole or paclitaxel (or equivalent amounts of DMSO for control) for 24 h. Then, culture medium was replaced by PBS containing 1 μg/ml propidium iodide (PI, for dead/injured cells labeling) and 5 μg/ml acridine orange (AO, to label all the cells) for 30 min. Cover slips were then taken for microscopic analyses on an ApoTome 2.0 (Zeiss) microscope adapted with a plan-apochromat 40 × 0.8 objective. HXP 120 C (metal halide) was used for illumination, and images were acquired from AO and PI labeled cells using excitation: BP 470/40; emission BP 525/50 and excitation: BP 550/25; emission BP 605/70, respectively. Zen software was used to collect images as .czi files using an AxioCam HRm camera.

### Estimation of *Brucella* CFU in Macrophages

Viable intracellular bacteria were estimated by counting CFUs. Infected macrophages were lysed for 10 min at room temperature in 800 μl of deionized water under manual agitation. Lysates were serially diluted from 10 to 10,000 times in PBS and plated on petri dishes containing Brucella Broth Agar. Petri dishes were incubated for 3 days at 37°C to allow proper growth of *Brucella* colonies to be counted as CFUs.

### Cytokine Analysis

Supernatant media from BMDMs infected and treated as described above were harvested 24 h after infection and assayed for the secretion of IL-1β, IL-12, IL-6 and TNF-α using ELISA kits (R&D Systems), in accordance with the manufacturer’s instructions.

### Statistical Analysis

GraphPad Prism computer software, version 6 (GraphPad Software) was used for analysis of the data. As indicated in the text, two-way ANOVA (followed by Bonferroni post-test) or Student’s *t*-test were performed. Levels of significance in the analysis were ^∗^*p* ≤ 0.05, ^∗∗^*p* ≤ 0.01 or ^∗∗∗^*p* ≤ 0.001 as indicated.

## Results

### Microtubule Alteration Affects Intracellular *Brucella* Replication

To investigate which aspects of *Brucella* intracellular infection are regulated by MTs, we induced disturbances into the cell cytoskeleton by chemical compounds or added the virulence factor TcpB and evaluated different aspects of host–pathogen interactions, such as: bacterial survival and replication, BCV structure and cytokine secretion. Initially, 0.1, 0.5, 1, 2, 5, and 10 μM of nocodazole (a MT depolymerizing agent) or paclitaxel (a MT stabilizer) were used (data not shown). Then, 0.5 and 5 μM concentrations were selected and cell viability was evaluated (Supplementary Figure 1). Treatment with 5 μM nocodazole for 24 h was the maximum concentration used to induce complete elimination of MT filaments without increase in cell death (Supplementary Figures 1A4,B). Similarly, incubation with 5 μM paclitaxel for 24 h promoted strong MT bundling (Supplementary Figures 1A5,B). Starting concentrations of 0.5 μM nocodazole and 0.5 μM paclitaxel were used in BMDM cells and also exhibited no effect on cell viability (Supplementary Figure 1A). Immunofluorescence analysis revealed that these lower concentrations of nocodazole and paclitaxel induced only minor alterations on the MT network (Supplementary Figure 1B).

**FIGURE 1:**
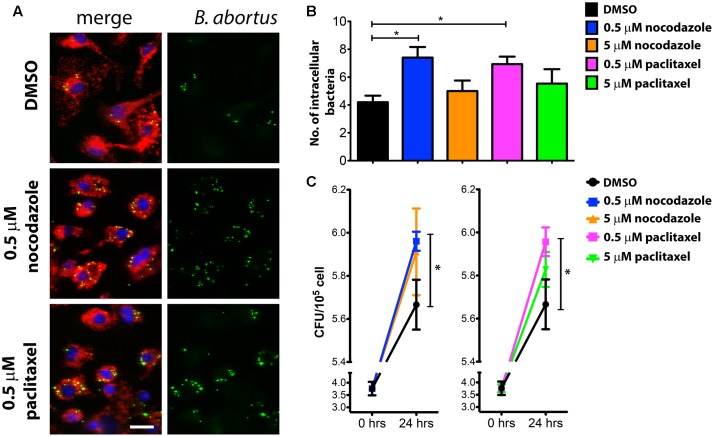
Intracellular bacterial load is increased in BMDMs treated with low doses of nocodazole or paclitaxel. BMDMs were infected with *B. abortus* and treated with nocodazole (0.5 or 5 μM), paclitaxel (0.5 or 5 μM), or vehicle (DMSO) for 24 h. Representative confocal micrographs of infected cells labeled with DAPI in blue and anti-LAMP1 in red are shown on the left **(A)**. GFP-expressing *Brucella* are shown in green. The number of GFP-expressing bacteria/cell was scored and is plotted in **(B)** as the average number of *Brucella*/macrophage. Data are expressed as mean ± SE and significant differences in relation to the DMSO control are designated by an asterisk (*p* ≤ 0.05, one-way ANOVA). Images are representative of three independent experiments and images from these three experiments were used to compute the graph shown in **(B)**. Number of cells evaluated per condition. DMSO (112), 0.5 μM nocodazole (124), 5 μM nocodazole (93), 0.5 μM paclitaxel (107) and 5 μM paclitaxel (85). Scale bar corresponds to 20 μm. Colony forming unit (CFU) analysis were performed at 0 and 24 hours post-infection (hpi) and the results are plotted in **(C)**. Data are expressed as mean ± SE of CFU counts obtained in triplicate in two independent experiments. Significant differences in relation to the DMSO control are designated by an asterisk (*p* ≤ 0.05, one-way ANOVA).

**FIGURE 2:**
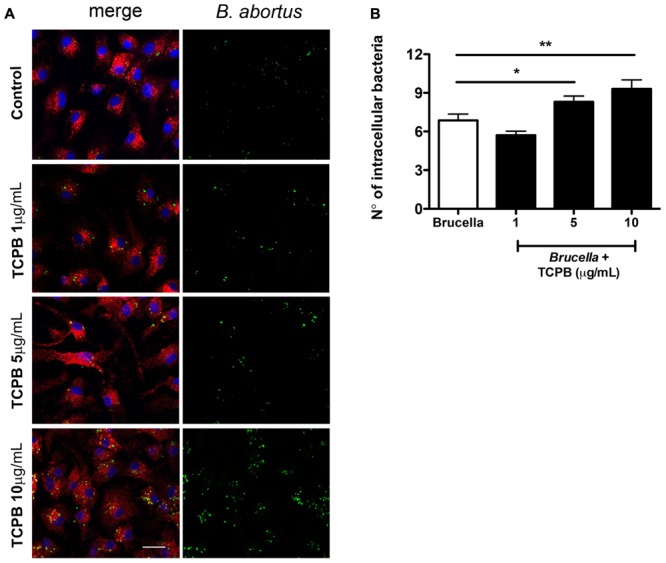
Intracellular bacterial load is increased in BMDMs treated with rTcpB protein. BMDMs were pre-treated with TCPB protein (1, 5, or 10 μg/mL) for 2 h and infected with *B. abortus* for 24 h. Representative confocal micrographs of infected cells are shown on the left **(A)** labeled with DAPI in blue and anti-LAMP1 in red. GFP-expressing *Brucella* are shown in green. The number of GFP-expressing bacteria/cell was scored and is plotted in **(B)** as the average number of *Brucella*/macrophage. Data are expressed as mean ± SE and significant differences in relation to control are designated by ^∗^*p* < 0.05 and ^∗∗^*p* < 0.01, (one-way ANOVA). Images are representative of three independent experiments. At least 85 cells were evaluated per condition. Scale bar corresponds to 20 μm.

*Brucella* survival and replication was then evaluated at 24 hours post-infection (hpi) in BMDMs treated with either 0.5 or 5 μM of nocodazole and 0.5 or 5 μM paclitaxel. To ensure that the disruptions on the MT cytoskeleton would not compromise bacteria internalization, macrophages were infected with *Brucella* (MOI 200:1) for 60 min in the absence of drugs. Cells were then washed to remove media containing extracellular *Brucella*, and cultured in medium containing DMSO (vehicle), nocodazole or paclitaxel for the remainder of the experiment. Bacterial load was assessed at 24 hpi by fluorescence microscopy (**Figure 1A**). Unexpectedly, strong MT disassembly or high MT stabilization in infected macrophages did not affect the intracellular bacterial load at 24 hpi compared to DMSO treated controls (**Figure 1B**). Conversely, treatment with 0.5 μM nocodazole and 0.5 μM paclitaxel promoted bacterial replication (**Figures 1A,B**). Despite intense GFP expression observed in imaged intracellular *Brucella*, used as an indication of live bacteria, CFU counts were also performed in infected macrophages to evaluate viability of the intracellular pathogen. In agreement with the immunofluorescence data, treatment with higher concentrations (5 μM) of nocodazole and paclitaxel had no effect on the viability of intracellular bacteria, whereas treatment with low concentrations of the same drugs promoted bacterial proliferation (**Figure 1C**). These results indicate that slight alterations in MT dynamics affect *Brucella* replication in BMDMs. Since TcpB also alters MT dynamics (Radhakrishnan et al., 2011), we treated infected cells with rTcpB and evaluated whether this molecule would interfere with *Brucella* survival or replication. *Brucella* growth was evaluated at 24 hpi in BMDMs pre-treated with different concentrations of rTcpB (1, 5, or 10 μg/mL) and bacterial load was assessed by microscopy. Interestingly, treatment with rTcpB promoted an increase in intracellular bacterial load in BMDMs infected with *B. abortus* (**Figures 2A,B**).

### Late BCV Composition Is Altered by Modifications of MT Dynamics

**FIGURE 3:**
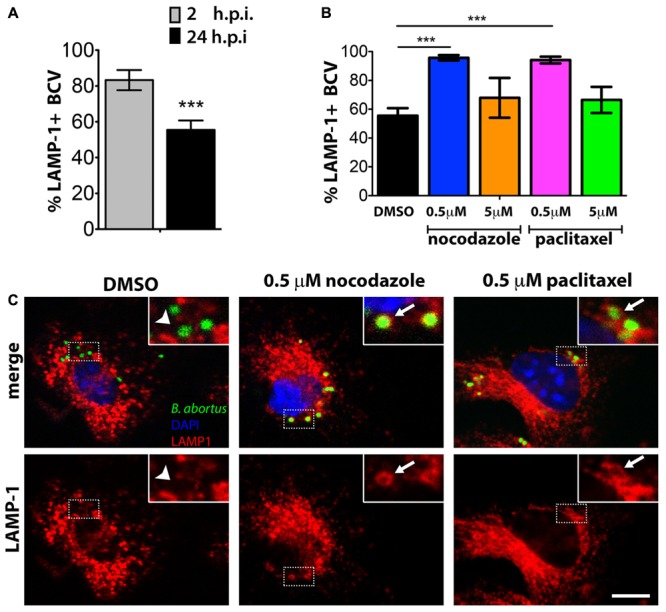
Association of Lamp-1 to late *Brucella*-containing vesicles (BCVs) are increased in BMDMs treated with low doses of nocodazole or paclitaxel. BMDMs were infected with *B. abortus* and treated with nocodazole (0.5 or 5 μM), paclitaxel (0.5 or 5 μM), or vehicle (DMSO) for 24 h. The percentage of LAMP1-positive BCVs was scored per cell, in control cells (DMSO) at 2 and 24 hpi **(A)**. For nocodazole and paclitaxel treated cells, LAMP1-positive BCVs were determined at 24 hpi **(B)**. Data are mean ± SE of BCVs showing LAMP-1 labeling per cell. Significant differences in relation to the DMSO control are designated by ^∗∗∗^*p* < 0.001 (one-way ANOVA). Representative confocal micrographs of BMDMs infected with *Brucella* and treated with DMSO, 0.5 μM nocodazole, or 0.5 μM paclitaxel for 24 h are shown in **(C)**. GFP-expressing *Brucella* are shown in green and macrophages were labeled with DAPI in blue and anti-LAMP1 in red on the merge panels. LAMP-1-positive BCVs are exemplified by arrows in inserts of the middle and left panels. BCVs showing weak or no association with LAMP-1 are shown on the right panels (arrow head). Inserts correspond to zoomed images (2.5 times amplification) of boxed areas. Scale bar corresponds to 5 μm.

**FIGURE 4:**
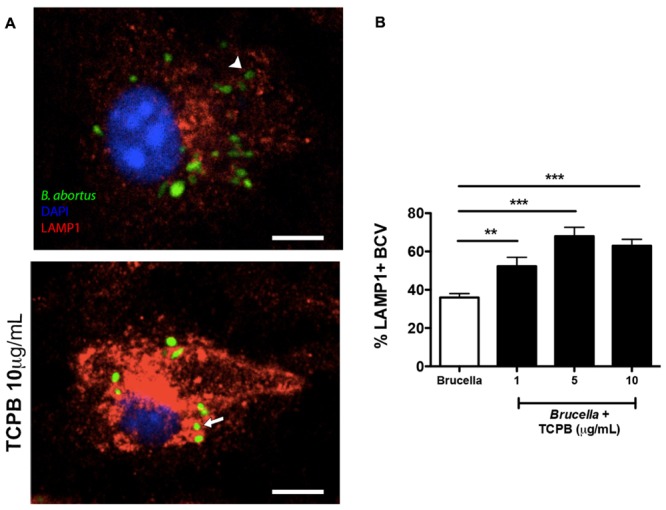
TcpB treatment enhances Lamp-1^+^ marker in late BCVs in infected BMDMs. BMDMs were pre-treated with rTcpB protein (1, 5, or 10 μg/mL) for 2 h and infected with *B. abortus* for 24 h. Representative confocal micrographs of BMDMs infected with *Brucella* and treated with rTcpB 10 μg/mL are shown in **(A)**. GFP-expressing *Brucella* are shown in green and macrophages were labeled with DAPI in blue and anti-LAMP1 in red. LAMP-1-positive BCVs are exemplified by arrows and BCVs showing weak or no association with LAMP-1 are exemplified by arrowheads. Scale bar corresponds to 5 μm. **(B)** The percentage of LAMP1-positive BCVs was scored per cell 24 hpi. Data are means ± SE of BCVs showing LAMP-1 labeling per cell. Significant differences in relation to the control are designated by ^∗∗^*p* ≤ 0.01 and ^∗∗∗^*p* ≤ 0.001 (one-way ANOVA).

The increased number of intracellular *Brucella* in BMDMs treated with 0.5 μM nocodazole or 0.5 μM paclitaxel could be an indication of alterations on BCV trafficking and/or maturation. To evaluate the effect of nocodazole and paclitaxel on BCV composition, we quantified the number of BCVs associated with the late BCV marker, LAMP-1 at 24 hpi, in treated and untreated infected macrophages. Two hours after infection, LAMP-1 association can be found in approximately 83% of BCVs (**Figure 3A**). Similarly, treatment of infected macrophages with 5 μM nocodazole or 5 μM paclitaxel showed no significant differences in percentage of LAMP-1-positive BCVs compared to untreated cells (**Figure 3B**). However, treatment of infected macrophages with 0.5 μM of nocodazole or 0.5 μM of paclitaxel, which cause weaker disturbances of the MT cytoskeleton, induced a dramatic change in LAMP-1 association with late BCVs. Either treatment caused a significant increase (to more than 90%) of LAMP-1 positive BCVs (**Figures 3B,C**). Similar experiments were performed to evaluate whether treatment with rTcpB would also have any effects on BCV trafficking. At 24 hpi, the number of late BCVs marked with LAMP-1 in untreated and rTcpB-treated infected macrophages was quantified. Treatment of infected macrophages with all studied concentrations of rTcpB induced a dramatic increase in the percentage of BCVs associated with the LAMP-1 marker (**Figure 4**). Our data support a correlation between TcpB-induced MT stabilization and the regulation of BCV composition during *B. abortus* infection, suggesting a crucial role of TcpB in the maturation of the BCV.

### IL-12 and IL-1β Secretion Induced by *Brucella* Infection in Macrophages Are Regulated by TcpB and Microtubule Network

**FIGURE 5:**
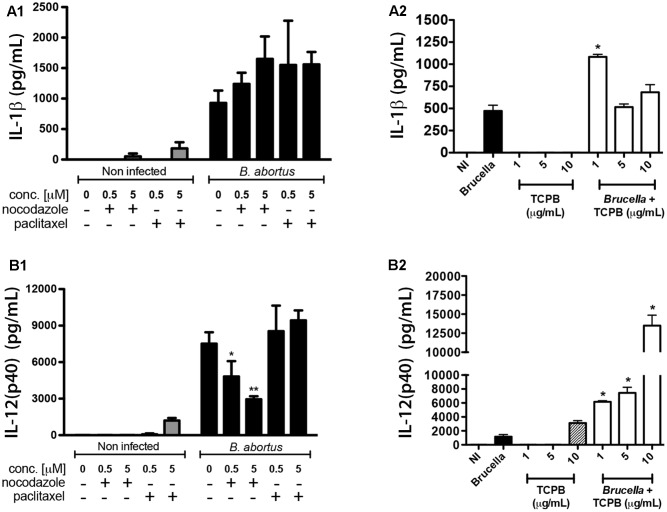
*Brucella*-induced pro-inflammatory cytokines are compromised by alterations on MTs dynamics. Expression of IL-12 **(A1,A2)** and IL-1β **(B1,B2)** was evaluated in control (uninfected) and *B. abortus*-infected BMDMs treated with nocodazole (0.5 or 5 μM), paclitaxel (0.5 or 5 μM), rTcpB (1, 5, or 10 μg/mL), or vehicle (DMSO). Supernatants were harvested after 24 h of stimulation and cytokine secretion was determined by ELISA. Significant differences in relation to the infected untreated controls are denoted (^∗^*p* ≤ 0.05; ^∗∗^*p* ≤ 0.01, one-way ANOVA).

Staying inside vesicular compartments throughout most of their intracellular life is one of the strategies developed by stealthy pathogens to hide from cytoplasmic innate immune receptors. However, endosomal receptors such as TLR3, TLR7-8 and TLR9, also take part in *Brucella* recognition (Gomes et al., 2016; Campos et al., 2017; Milillo et al., 2017) and likely encounter BCVs during bacterial intracellular trafficking. Since vesicular traffic is largely performed by MTs, we investigated whether treatment of infected macrophages with nocodazole, paclitaxel or rTcpB could also affect innate immune responses against *Brucella* infection. Secretion of IL-1β, IL-12, IL-6 and TNF-α was measured in untreated as well as drug-treated uninfected macrophages to evaluate any effects of MT disturbance on basal levels of cytokine production. Although MT destabilization with either 0.5 or 5 μM nocodazole and stabilization with 5 μM paclitaxel were sufficient to stimulate IL-6 and TNF-α secretion in uninfected BMDM cells (Supplementary Figures 2A,B), their levels were not significantly different in treated versus untreated *B. abortus* infected macrophages. Also, no difference on secretion of IL-1β was observed in untreated or drug-treated macrophages infected with *B. abortus* (**Figure 5A1**). In contrast, treatment with the lower concentration of rTcpB (1 μg/ml) in infected macrophages promoted a significant increase in IL-1β secretion (**Figure 5A2**). Interestingly, IL-12 production induced by *B. abortus* infection was significantly affected by nocodazole treatment in a dose-dependent manner and a decrease of almost 60% in cytokine secretion was observed in BMDMs treated with 5 μM nocodazole (**Figure 5B1**). In contrast, rTcpB significantly enhanced IL-12 secretion in treated macrophages infected with *B. abortus*, in a dose-dependent fashion (**Figure 5B2**). Additionally, treatment of infected macrophages with either 0.5 or 5 μM paclitaxel had no effect on IL-12 secretion. These results indicate that decrease in IL-12 secretion observed in nocodazole-treated infected macrophages was related to MT destabilization, and the inverse effect promoted by TcpB coincides with the described feature of this protein as a MT stabilizing agent. In summary, our findings suggest a relevant role for MT dynamics in innate immune responses triggered by *B. abortus* infection.

## Discussion

Due to the regulatory roles played by MTs on virtually all cellular processes, it is no surprise that intracellular pathogens, such as *Brucella*, have evolved to take advantage of this cytoskeleton in some of their survival and replication strategies (Radhakrishnan and Splitter, 2012). The intricate network of MT filaments is the main pathway on which vesicles, organelles and different cargos are moved, via molecular motor-mediated transport, inside interphase cells. To survive and replicate inside phagocytic cells, *Brucella* needs to orchestrate structural changes in the vacuoles. One major modification observed during maturation from early to late replicative-BCVs (eBCV to rBCV) is the elimination, over time, of the LAMP-1 marker acquired soon after *Brucella* invades macrophages (Starr et al., 2008; von Bargen et al., 2012; de Souza Filho et al., 2015). However, removal of LAMP-1 from intermediate BCVs is not a requirement for their maturation into rBCVs, since replication of *B. abortus* has been observed in LAMP-1 positive vesicles in THP1 cells and murine macrophages (Bellaire et al., 2005; Starr et al., 2012). Therefore, intermediate steps of BCV trafficking and maturation are still far from being understood. Our results indicate that manipulation of MT dynamics toward the increase of either MT depolymerization or polymerization events at their ends, interfere with BCV maturation and replication of *B. abortus*. However, further analysis with additional markers of different endosomal, Golgi- and ER-derived compartments are necessary to fully characterize the rBCV in nocodazole and paclitaxel treated macrophages. Recently, nocodazole treatment has been shown to interfere with the first steps of *B. abortus* infection in RAW 264.7 cells as pre-treatment of RAW cells with 0.4 μg/ml nocodazole increased adhesion of *Brucella* to the host cell. However, despite the increase in cell attachment, invasion and intracellular growth of bacteria was reduced when compared to control cells (Reyes et al., 2017). Additionally, no alterations were found in the percentage of *Brucella* co-localization with LAMP-1^+^ compartments between control and treated cells. The contrasting results between the Reyes et al. (2017) study and ours may reside in the fact they used a different strain of *B. abortus* (*B. abortus* 544), as well as performing their experiments using the RAW 264.7 cell line and not BMDMs.

From invasion to dissemination, all stages of the intracellular bacterial life cycle share the same three-dimensional cytosolic space containing the host cytoskeleton. For successful infection and replication, many pathogens hijack the cytoskeleton using effector proteins introduced into the host cytosol by specialized secretion systems (Colonne et al., 2016). Cytoskeletal rearrangement promotes numerous events that are beneficial to the pathogen, including internalization of bacteria, structural support for bacteria-containing vacuoles, altered vesicular trafficking, actin-dependent bacterial movement, and pathogen dissemination. The stealth pathogen *Brucella* is renowned for its silent entry into the host and avoidance of phagolysosomal degradation, and the translocated protein TcpB has been shown to contribute to this strategy by targeting the TLR-dependent arm of the host innate immune defense (Alaidarous et al., 2014). However, the situation seems more complex, as TcpB is also involved in MT dynamics and, recently, in the induction of the unfolded protein response (Radhakrishnan et al., 2011; Smith et al., 2013). In this study, treatment with TcpB causes an increase in intracellular bacterial growth in macrophages that corroborates previous *in vivo* mouse studies that implied TcpB-deficient *Brucella* as defective in systemic spread at early stages of infection (Radhakrishnan et al., 2009). Likewise, *Brucella melitensis* can induce an Unfolded Protein Response via TcpB that supports intracellular growth in macrophages (Smith et al., 2013). Increase in LAMP-1^+^ BCVs treated with rTcpB, suggests that the MT bundling generated by TcpB interferes with the vesicular transport along the MT network to the benefit of pathogen survival.

Pro-inflammatory cytokine production by innate immune cells is critical in the process of host control of intracellular pathogens. Herein, we demonstrated that treatment with nocodazole lead to reduced IL-12 production, however, no alteration was observed in IL-1β, IL-6 and TNF-α. Recently, Reyes et al. (2017) reported that *Brucella*-infected nocodazole -treated mice presented higher levels of TNF, IFN-γ, MCP-1, IL-10 and IL-6 as compared to control untreated animals. Interestingly, although not mentioned in their study, their data would suggest that *Brucella*-induced IL-12 expression was robustly reduced by nocodazole treatment as several of these cytokines are dependent on IL-12, a result which corroborates with our findings. Moreover, depolymerization of the MT network disrupted intracellular TLR2 and TLR4 and inhibited IL-12 production in response to *Neisseria meningitidis* infection (Uronen-Hansson et al., 2004). These results suggest that the TLR activation by *N. meningitidis* required for IL-12 production occurs inside DCs and not on the cell surface. We have extensively reported that TLR9 senses *Brucella* CpG to induce IL-12 production (Macedo et al., 2008; Gomes et al., 2016). Therefore, intracellularly expressed TLR9 is likely affected by interference with MT dynamics compromising IL-12 secretion.

TcpB also interferes with innate immune responses following *B. abortus* infection. Subcellular colocalization studies indicate that TcpB colocalizes with the plasma membrane and MTs (Radhakrishnan et al., 2009). Further, the same authors also reported that TcpB affects the dynamics of MT formation stabilizing polymerized tubules (Radhakrishnan et al., 2011). In contrast to nocodazole treatment, rTcpB enhanced IL-12 production in a dose-dependent manner. Additionally, rTcpB-treated macrophages (1 μg/ml) augmented IL-1β secretion after *Brucella* infection. Taking into account that TcpB targets the TIRAP-mediated pathway, leading to subversion of TLR2 and TLR4 signaling and inhibition of NF-κB (Salcedo et al., 2008; Radhakrishnan et al., 2009), IL-12 would be expected to decrease in the presence of rTcpB. However, we and others have demonstrated that TLR9 and not TLR2 and TLR4 is the main receptor involved in IL-12 production by *Brucella* (Giambartolomei et al., 2004; Macedo et al., 2008; Gomes et al., 2016). Furthermore, Salcedo et al. (2013) have reported that TcpB does not inhibit TLR9. Therefore, further studies are necessary to unravel which innate immune pathways are related specifically with TcpB modulation of MTs that affects cytokine production. Detailed investigation on MT–pathogen interactions will provide broad understanding of pathogenicity and host adaptation of several infectious pathogens.

In summary, this study reveals that modulation of MTs affects several crucial steps of *B. abortus* pathogenesis, including BCV maturation, intracellular survival, and IL-12 secretion in infected macrophages. MTs are potential targets of pathogenic microorganisms to create a replication-permissive niche. Therefore, our results reflect an important relationship between an intracellular bacterial pathogen and MTs that contributes to our understanding of *B. abortus* pathogenicity.

## Author Contributions

JA-S, JM, GS, and SO designed the project and experiments. JA-S, IT, EG, and BF carried out most of the experiments. JA-S and SO wrote the manuscript. JA-S and EG carried out statistical analysis and prepared figures. SO submitted this paper. All authors reviewed the manuscript.

## Conflict of Interest Statement

The authors declare that the research was conducted in the absence of any commercial or financial relationships that could be construed as a potential conflict of interest.

## Footnotes

1 https://www.dna20.com
