# Supplementary material for: Modulation of Microtubule Dynamics Affects Brucella abortus Intracellular Survival, Pathogen-Containing Vacuole Maturation, and Pro-inflammatory Cytokine Production in Infected Macrophages
Source: Front Microbiol. 2017 Nov 14;8:2217. doi: 10.3389/fmicb.2017.02217 (PMC5694624; doi:10.3389/fmicb.2017.02217)
Supplement: Supplementary file 1 [file Image_1.pdf]

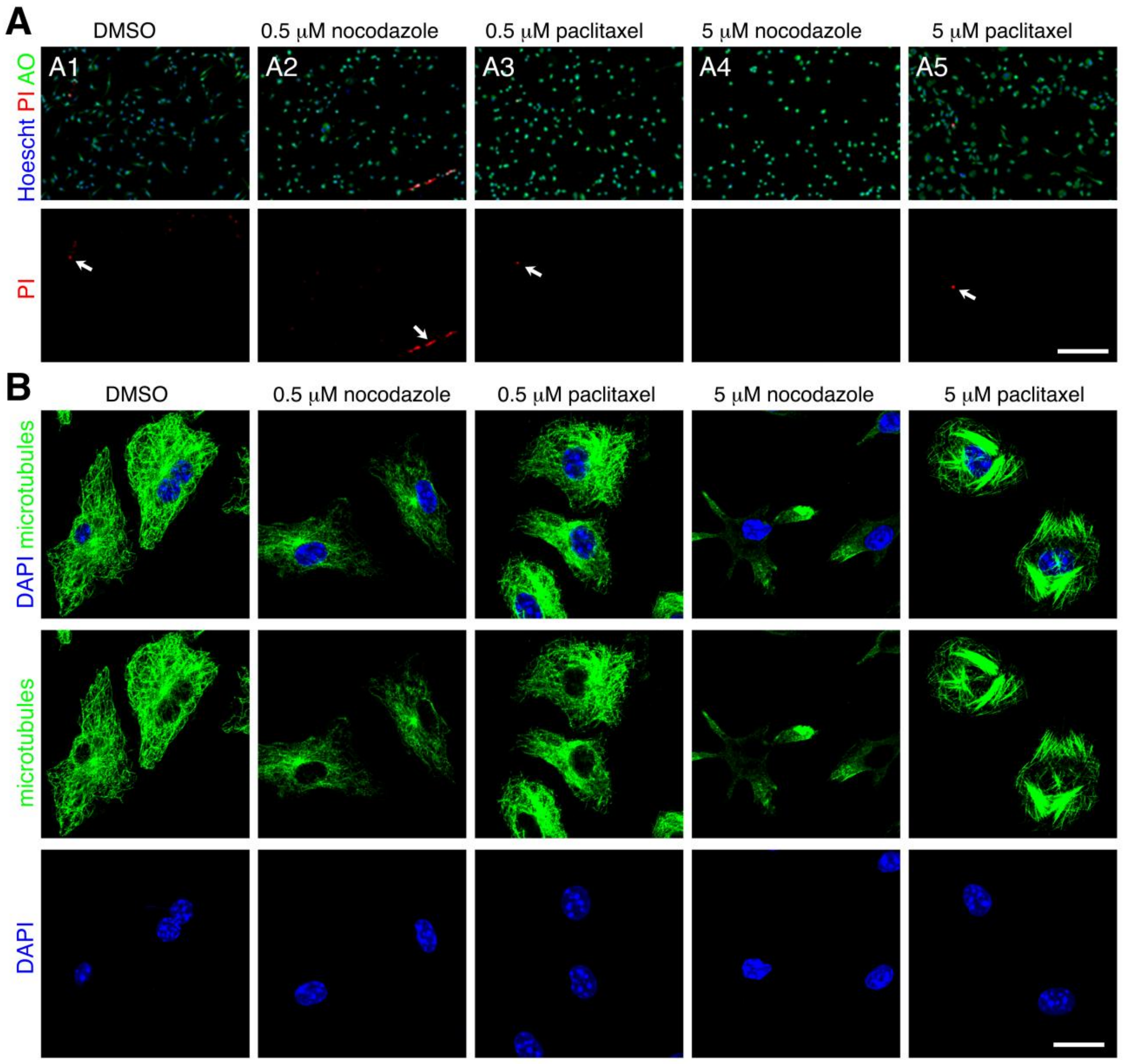

Supplementary figure 1. Nocodazole and paclitaxel treatments have no effect on BMDM survival.

BMDMs were treated for 24 hours with 0.5 μM nocodazole (A2), 0.5 μM paclitaxel (A3), 5 μM nocodazole (A4), 5 μM paclitaxel (A5), or vehicle (A1, DMSO) and evaluated for cell viability. Panels in A show representative confocal micrographs of cells labeled with DAPI in blue and acridine orange in green. Dead cells are labelled with propidium iodine (arrows in A1, A2, A3 and A5). Panels in B show fixed cells from the same experiments, in which the microtubules were labelled in green with anti-tubulin to demonstrate the effect of different doses of nocodazole and paclitaxel (indicated above in each column). Cells nuclei are labelled with DAPI (in blue). Images are representative of three independent experiments. Scale bars correspond to 100 μm in A and 15 μm in B.

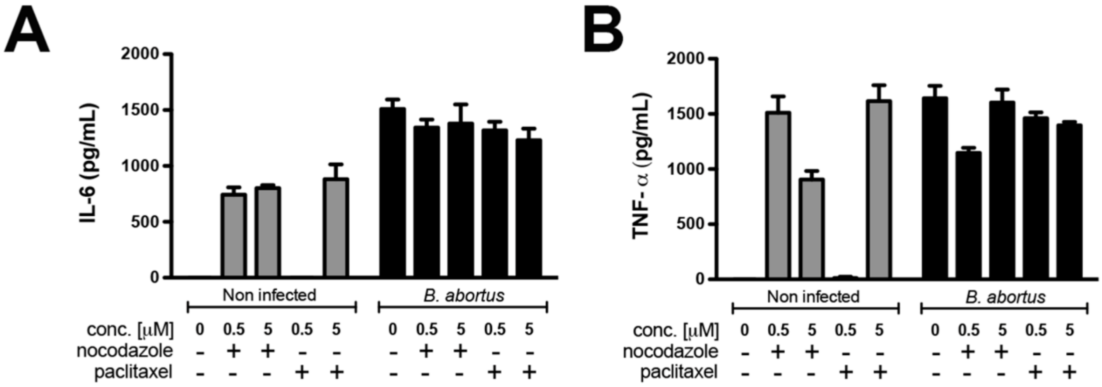

Supplementary figure 2. Nocodazole and paclitaxel treatment stimulates IL-6 and TNF-α expression in BMDMs. Expression of IL-6 (A) and TNF-α (B) was evaluated in control (uninfected) and *B. abortus*-infected BMDMs treated with nocodazole (0.5 or 5 μM), paclitaxel (0.5 or 5 μM), or vehicle (DMSO). Supernatants were harvested after 24 hours of stimulation and cytokine secretion was determined by ELISA.
